# *Nocardia* scleritis—clinical presentation and management: a report of three cases and review of literature

**DOI:** 10.1007/s12348-011-0043-9

**Published:** 2011-10-08

**Authors:** Srikant Kumar Sahu, Savitri Sharma, Sujata Das

**Affiliations:** 1 Cornea and Anterior segment Services, Proff Brien Holden Eye Research Center, L V Prasad Eye Institute, Bhubaneswar, Patia, Bhubaneswar, Orissa 751024 India; 2 Ocular Microbiology service, Proff Brien Holden Eye Research Center, L V Prasad Eye Institute, Bhubaneswar, Patia, Bhubaneswar, Orissa India

**Keywords:** Scleritis, *Nocardia*, Amikacin

## Abstract

**Aim:**

This study aims to describe the clinical features and management of *Nocardia* scleritis.

**Methods:**

The authors retrospectively reviewed medical charts of three patients with microbiologically proven *Nocardia* scleritis and reviewed literature.

**Results:**

All the patients presented with areas of well-demarcated, circumscribed abscess. No specific clinical feature could be attributed to the causative organism. *Nocardia* was identified by smear and culture from the scleral exudates. The medical management was based on the antibiotic sensitivity. Surgical exploration of the suppurated area along with the healthy margins was done on all patients. Two patients required multiple explorations. All three patients resolved with a good visual and tectonic outcome. The literature review also suggests a good outcome with prolonged medical management though the preferred antibiotic has changed over the years.

**Conclusion:**

Though the prevalence of a disease like *Nocardia* scleritis is low, the results suggest that specific diagnosis and appropriate management can lead to a good outcome.

## Introduction

Isolated *Nocardia* scleritis is rare, and usually occurs due to an extension of corneal infection involving the limbus [1]. The available literature is mostly a collection of case reports [2–11]. It is difficult to diagnose due to its rarity. A delay in diagnosis and specific treatment can lead to potential sight-threatening complications. Intraocular extension of Nocardial scleritis has been reported [10]. Although there are several reports on *Nocardia* keratitis and sclerokeratitis in the literature (Pubmed), there are few published data on isolated *Nocardia* scleritis [12, 13]. We report the clinical presentation and management of three cases of isolated scleritis due to *Nocardia* spp. We have also reviewed all the cases published till date.

### Case 1

A 65-year-old man complained of a white mass on the lower conjunctiva that had formed since 2 months associated with pain and redness in the right eye. His right eye vision was 20/100. He was receiving topical gatifloxacin eye drop four times per day, tobramycin eye drop every hour, and oral analgesic twice daily. On examination the lower fornix had localized circumscribed area of scleral abscess with engorged episcleral and conjunctival vessel around it. Smear and culture revealed *Nocardia* species, sensitive to amikacin (Table 1). The patient was initiated on topical amikacin 2.5% every half hour and oral trimethoprim (160 mg)–sulfhamethoxazole (800 mg), twice daily. The abscess persisted despite treatment with culture adjusted antibiotics for 6 weeks. Hence we explored the sclera to express out purulent material. Postoperatively topical gatifloxacin every hour was added to previous treatment. The lesion persisted for the next 3 weeks and hence the abscess was again re-explored. Apart from the infected area a thorough cleaning of healthy margin was done looking specifically at any extension to the surrounding area. Postoperatively we continued the same medications. Systemic antibiotic were continued for 2 months. At fourth month of treatment his vision was 20/60 and there was granulation tissue at the lesion site. A slit lamp examination revealed that he had nuclear sclerosis.

**Table 1:** Clinical summary, management, and outcome of all reported cases of isilated *Nocardia* scleritis

Sl no.	Author	Eye	Age	Sex	Predisposing factor	Duration of symptoms	Presenting VA	Final VA	Treatment—medical	Treatment—surgical	Outcome
1	Kattan [3]	OD	63	F	Scleral buckle, cataract surgery	2 weeks	1/200	NA	Topical and TMP-SMX (iv)	Eviseration	Eviseration
2	King [5]	OD	77	M	Scleral buckle, cataract surgery	14 weeks	20/60	20/40	Topical 10% sulphacetamide, TMP-SMX (iv)	Buckle removal	Resolved
3	Brooks [4]	OS	90	F	Cataract surgery	5 weeks	20/80	NA	Topical amikacin, topical and oral TMP-SMX	Surgical debridement, scleral patch graft	Resolved
4	Basti [2]	OD	58	M	Injury with vegetable material, steroids	1 month	20/200	20/40	Cefazolin (systemic and topical)	Nil	Resolved
5	Knox [8]	OS	83	M	Explantation of exposed buckle, steroids	NA	20/100	20/60	Topical amikacin 2.5%, TMP-SMX 160/800	Nil	Resolved
6	Choudhry [10]	OS	54	M	Steroids	7 Weeks	20/30	NA	Cefazolin (systemic and topical), TMP-SMX 160/800, 10% sulphacetamide	Surgical debridement	Worsened and lost to follow up
7	Das [9]	OS	40	F	Mud splash, steroids	7 weeks	20/20	20/20	Topical TMP-SMX 160/800, amikacin 2.5%, amoxycillin/clavulanate (800/125 mg)	Nil	Resolved
8	Seth [6]	OD	80	F	Subtenon steroid	3 months	20/400	CF 1 m	Topical trimetho prim/polymixin B, minocycline 100 mg/day	Scleral exploration and biopsy and bovine pericardium patch graft	Resolved
9	Jain [11]	OD-2 OS-1	60.66 ± 7.36	M-3	Scleral buckle—*n* = 2	1–2 months	20/125–20/400	20/80–20/400	Topical amikacin—all cases, TMP-SMX 160/800—*n* = 2	Debridement *n* = 1	Resolved—all
10	DeCroos [12]	OD-8 OS-3	56.8 ± 13	M-11	Injury with organic material—*n* = 5	30.4 ± 16.8 days	Mean BCVA—20/502	Mean BCVA—20/399	Topical amikacin/ciprofloxacin/cefazolin—*n* = 11	Surgical debridement—*n* = 8; conjunctival excision—*n* = 2; tissue adhesive—*n* = 1	Resolved
					Scleral buckle—*n* = 1				Systemic amikacin—*n* = 3		
					PPV—*n* = 1						
11	Maruo [13]	OS	78	M	Cataract surgery	2 months	20/30	20/16	Topical tobramycin, moxifloxacin, oral TMP-SMX	Debridement	Resolved

*OD* right eye, *OS* left eye, *NA* not available, *VA* visual acuity, *TMP-SMX* trimethoprim 160–800, *PPV* pars plana vitrectomy

### Case 2

**Fig. 1:**
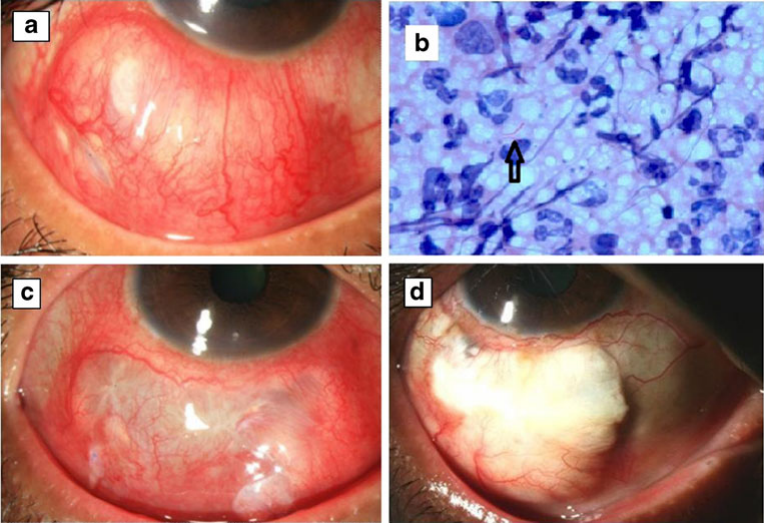
Patient no. 2. **a** Slit lamp picture showing an area of circumscribed scleral abscess. A scleral ulcer is situated inferior to the abscess. **b** Scleral scraping showing thin, branching, acid fast filaments (*arrow*) suggestive of *Nocardia* species (Kinyoun’s acid fast stain using 1% H_2_SO_4,_ ×1,000). **c** Three weeks after treatment decipitating uveal show. **d** Healthy and vascularized graft 4 months after scleral patch graft

A 50-year-old lady with history of injury to the right eye with some plant material 2 1/2 months ago developed pain, redness and watering. She was on topical levofloxacin and lubricant. Her vision was 20/30 in right eye and 20/20 in left eye. The right eye showed a well-localized, circumscribed abscess and a scleral ulcer discharging purulent material in the inferior bulbar conjunctival area (Fig. 1a).The abscess was drained and the surrounding necrotic tissue was removed. Smear and culture of the exudates were positive for *Nocardia* species (Fig. 1b). Topical amikacin 2.5% every hour, ciprofloxacin 0.3% every hour, homatropine twice daily, and oral ciprofloxacin 500 mg twice daily was given. After 3 weeks of treatment there was no area of scleral suppuration or ulcer, conjunctiva was mildly congested, and there was an area of uveal show (Fig. 1c) under the conjunctiva. A donor scleral patch graft with amniotic membrane graft over it was done. Postoperatively we advised topical prednisolone acetate eight times a day, ciprofloxacin 0.3% every hour, and oral ciprofloxacin 500 two times a day. The systemic medication was discontinued after a week, topical corticosteroids were tapered, and antibiotic was continued for 2 months. Four months postoperatively her best corrected visual acuity was 20/20 with a healthy graft.

### Case 3

A 55-year-old lady presented with a history of pain and redness in the left eye for 2 months. She was on oral prednisolone (1 mg/kg body weight), and topical chloramphenicol-dexamethasone for the last 2 months. Her vision in the left eye was 20/200. Slit lamp examination of the left eye showed a circumscribed abscess in the superior bulbar area and cataract. Scleral exudates showed thin acid fast branching filaments under microscope and culture revealed *Nocardia asteroides* (identified based on biochemical tests). She was initiated on topical amikacin 2.5% and ciprofloxacin 0.3% every hour and oral ciprofloxacin 500 mg twice daily. The sclera was explored twice because of recurrence in the inferotemporal quadrant. Postoperatively she was advised topical amikacin 2.5% and gatifloxacin 0.3% every hour along with systemic gatifloxacin 400 mg once daily. At 3 months follow-up the corrected visual acuity at 20/50; there was cataract, though the sclera was thinned and there was uveal show.

### Surgical technique

The conjunctiva overlying the suppurated area was excised. The suppurated material along with necrotic sclera was removed. These were sent for a microbiology evaluation. Care was taken to clean the margin as far as possible to obtain a healthy sclera. The base and margin were irrigated thoroughly. Postoperatively subconjunctival amikacin was injected to the surrounding conjunctiva. At the end of surgery the conjunctiva was not closed. This helps in the penetration of the topical medication

### Microbiology evaluation

The scleral scrapings/exudates were subjected to microscopic examination (Gram stain, modified Zeihl Neelsen stain using 1% H_2_SO_4_) and culture on sheep blood agar, chocolate agar, brain heart infusion broth, thioglycollate broth, and Sabouraud dextrose agar. Microscopic examination of the scleral exudates showed acid fast thin, beaded, branching filaments in all patients. In all cases, slow growing, chalky white dry colonies grew on blood and chocolate agar. Based on biochemical tests such as urease production, hydrolysis of casein, xanthine, tyrosin, and growth in gelatine, one of the isolates was identified as *N. asteroids.* (The susceptibility of the isolates was tested on Muller Hinton blood agar by disk diffusion method).

## Discussion

Isolated *Nocardia* scleritis is rare and usually occurs as an extension of corneal infection involving the limbus [1]. A clinical summary of all cases reported to date has been compiled in Table 2. The common predisposing factors were surgery and injury (scleral buckle—5, injury—7). Clinical features of *Nocardia* keratitis like patchy infiltrate, wreath-like pattern has been described [14]. No such clinical feature which is pathognomonic of any causative organism has been described in cases of scleritis. Sixteen of the 23 published cases had a history of injury or surgery which can lead to a compromised ocular surface. Two of three patients did not have any history of trauma or surgery, though one patient (no. 1) was an agriculturist by profession and trivial trauma is expected. With no specific clinical feature and history only a microbiological evaluation could lead us to a specific diagnosis.

**Table 2:** In vitro antibiotic susceptibility (by Kirby Bauer disk diffusion technique)

	Amikacin	Cefazolin	Chloramphenicol	Ciprofloxacin	Gatifloxacin	Ofloxacin	Vancomycin
Case 1	+	+	+	+	+	ND	ND
Case2	+	−	−	+	+	+	−
Case 3	+	−	+	−	+	−	+

+ sensitive, − resistant, *ND* not done

The lesions in 17 out of 23 patients had resolved with treatment. Topical amikacin 2.5%, TMP-SMX, cefazolin, sulphacetamide10%, and systemic TMP-SMX were the most common medication used. In vitro antibiotic sensitivity (by Kirby Bauer disk diffusion technique) of our patients is compiled in Table 1. The organisms were susceptible to amikacin and gatifloxacin in all cases whereas the organisms were resistant to cefazolin in two of the three cases. These findings are in concordance with DeCroos et al. [13] who describe maximum sensitivity to amikacin and gatifloxacin and minimal sensitivity to cefazolin. Intensive topical amikacin 2.5% along with a flouroquinolone was used for treatment in all our cases. Oral TMP-SMX was advised in one case and flouroquinolones in two cases. Sclera being an avascular structure, the bioavailability of systemic antibiotics is poor, therefore, the period of systemic antibiotic was prolonged from 4–8 weeks.

Out of 21 of the published cases who recovered, 17 needed surgical intervention. In the series described by DeCroos et al. [12], four of the 11 required multiple interventions. All three of our patients were debrided and exudative materials were removed till healthy sclera was seen. Patients no. 1 and no. 3 were explored multiple times. This can be explained by the presence of tunnel lesion as described by Lin et al. [15] and Raber et al. [16] in patients with infectious scleritis. These lesions extend only into the deep layers of sclera with the superficial sclera and conjunctiva being infection free. Thus even after good cleaning of margins the infection tends to recur. One patient required a scleral patch graft after the primary lesion had resolved as there was a large area of uveal show.

Cataract was the cause of decreased vision in two of our patients. Uveitis, choroidal detachment, and intraocular spread of organism are other complications described in the literature [3, 12].

In conclusion, a complete microbiological workup leads to an early and specific diagnosis. Along with intensive, prolonged topical and systemic medication; surgical debridement of the necrotic material is necessary for complete resolution of the disease.
